# Epigenetic Regulation of ITGB7 Promotes Coronary Heart Disease via Immune and Metabolic Pathways: A Multimodal Mendelian Randomization Study

**DOI:** 10.1155/cdr/4885476

**Published:** 2026-01-07

**Authors:** Junchi Guo, Fang Huang, Peihan Lu, Meijuan Lu

**Affiliations:** ^1^ Department of Graduate School, Tianjin University of Traditional Chinese Medicine, Tianjin City, China, tjutcm.edu.cn; ^2^ Department of Cardiology, Second Affiliated Hospital of Tianjin University of Traditional Chinese Medicine, Tianjin City, China, tjutcm.edu.cn

**Keywords:** CHD, DNA methylation, ITGB7, Mendelian randomization

## Abstract

**Background:**

Coronary heart disease (CHD) is a leading cause of cardiovascular mortality worldwide, with its pathogenesis being complex and not yet fully understood. The rapid development of genomics, especially in epigenetic research, has provided essential tools for identifying new pathogenic targets. This study is aimed at systematically exploring the molecular mechanisms of CHD using protein quantitative trait locus (pQTL) data and multiomics Mendelian randomization (MR) approaches, with a specific focus on the epigenetic regulation of the key gene ITGB7.

**Methods:**

The study first integrated 1812 *cis*‐pQTL data with CHD GWAS data to perform a two‐sample MR analysis, identifying protein‐coding genes significantly associated with CHD. Transcriptomic data were then used to validate the differential expression of these genes. Subsequently, a two‐step MR mediation analysis was conducted to explore the upstream regulatory effect of DNA methylation on the key gene ITGB7, as well as the potential mediating roles of ITGB7 on downstream immune cells and plasma metabolites.

**Results:**

MR analysis identified 17 genes significantly positively associated with CHD, with PCSK9 and ITGB7 showing significant upregulation in the peripheral blood of CHD patients. Mediation analysis revealed that the DNA methylation site cg14524975 (beta_*p* = 45.64*%*) significantly increased the risk of CHD by positively regulating the expression of ITGB7. In downstream mechanisms, ITGB7 significantly promoted CHD progression by regulating immune cells, such as CD4+ CD8dim AC (beta_*p* = 12.04*%*), and plasma metabolites, including *N*,*N*‐dimethylalanine (beta_*p* = 18.96*%*), benzoate‐to‐oleoyl–linoleoyl–glycerol (18:1 to 18:2) ratio (beta_*p* = 34.63*%*), and serine‐to‐threonine ratio (beta_*p* = 12.58*%*).

**Conclusion:**

This study identifies ITGB7 as a novel pathogenic gene for CHD and reveals its multiomics mechanisms in promoting CHD development through DNA methylation regulation, immune response activation, and metabolic pathway disruption. The findings provide valuable theoretical insights and potential biomarkers for the pathogenesis and targeted intervention of CHD.

## 1. Introduction

Coronary heart disease (CHD) is one of the leading causes of morbidity and mortality worldwide, accounting for more than one‐third of all cardiovascular deaths each year [1]. The large‐scale epidemiological study INTERASPIRE has shown that, although modern pharmacological treatments and interventional techniques have significantly improved the prognosis of CHD patients, their incidence and recurrence rates remain high—particularly among high‐risk populations [2]. This suggests that, beyond traditional risk factors such as dyslipidemia, hypertension, and diabetes, further exploration of novel pathogenic mechanisms and potential therapeutic targets for CHD is urgently needed [3].

Compared with traditional observational studies, the Mendelian randomization (MR) approach can effectively reduce confounding bias and reverse causation, thereby providing more reliable evidence for elucidating disease mechanisms and identifying potential therapeutic targets [4]. In particular, the integration of protein quantitative trait locus (pQTL) data enables researchers to identify potential biomarkers and therapeutic targets at the protein level [5], opening new avenues for targeted therapy of CHD. A well‐established example is proprotein convertase subtilisin/kexin type 9 (PCSK9), a gene strongly associated with low‐density lipoprotein cholesterol (LDL‐C) levels [6]. The clinical application of PCSK9 inhibitors has significantly reduced the occurrence of cardiovascular events, demonstrating the great potential of gene‐based targeted therapy in cardiovascular diseases [7]. The success of PCSK9 provides a valuable paradigm illustrating how in‐depth genomic exploration can uncover novel therapeutic targets for CHD. However, the currently identified genes explain only part of the genetic susceptibility to CHD, while other underlying molecular mechanisms—particularly epigenetic regulation and gene–environment interactions—remain insufficiently investigated [8]. Moreover, existing pQTL studies are mostly confined to single‐omics analyses at the protein level and have not systematically considered upstream epigenetic regulatory factors or downstream biological consequences.

The UK Biobank Pharma Proteomics Project (UKB‐PPP) is a large‐scale proteomics study based on samples from the UK Biobank, providing quantitative data on plasma proteins [9]. This study is aimed at identifying novel CHD targets by conducting MR analyses using UKB pQTL data. Furthermore, we intend to investigate upstream epigenetic regulation via DNA methylation and downstream changes in immune cell profiles and plasma metabolites for key genes. Through this multilayered, multiomics strategy, we hope to provide new insights and directions for the precision treatment of CHD.

## 2. Methods

**Figure 1:**
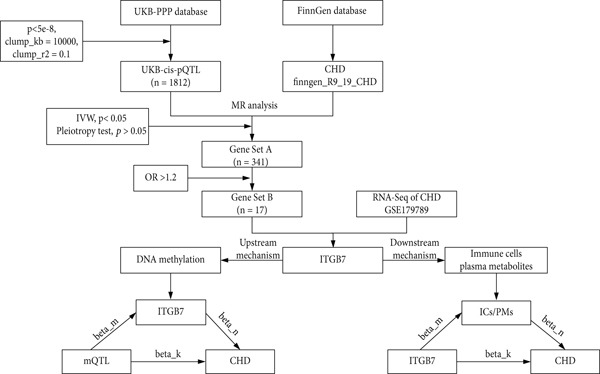
Detailed workflow of the current study.

All data used in this study were obtained from publicly available international databases, adhering to relevant usage guidelines and therefore not requiring additional ethical approval. A detailed flowchart of the study design is shown in Figure 1.

### 2.1. *cis*‐pQTL Data

The raw pQTL data were downloaded from the UKB‐PPP database (https://www.synapse.org/Synapse:syn51364943/wiki/622119/) [9]. Based on the criteria that single‐nucleotide polymorphisms (SNPs) and their corresponding genes are located on the same chromosome and within ± 1 Mb of each gene, *cis*‐pQTLs were extracted, resulting in a total of 2918 *cis*‐pQTL files. These were filtered using the following parameters: *p* < 5e − 8, clump_kb = 10,000, and clump_*r*2 = 0.1, yielding *cis*‐pQTL data for 1812 protein‐coding genes [10].

### 2.2. CHD Data

Genome‐wide association study (GWAS) summary data for CHD were downloaded from the FinnGen database (https://www.finngen.fi/en/), specifically dataset ID finngen_R9_I9_CHD. This dataset includes a total of 377,277 participants, comprising 43,518 CHD cases and 333,759 controls. Among the CHD cases, 12,521 were female (prevalence: 5.94%; mean age of onset: 68 years), and 30,997 were male (prevalence: 18.64%; mean age of onset: 65.16 years).

### 2.3. MR Analysis

In this study, *cis*‐pQTLs for 1812 genes were used as exposures and CHD as the outcome. Weak instrumental variables with *F* statistics < 10 were excluded. Two‐sample MR was conducted using the TwoSampleMR package. The inverse variance weighted (IVW) method was applied as the primary analytical model, and genes with a *p* value < 0.05 were considered significantly associated with CHD. Horizontal pleiotropy was tested using the MR–Egger intercept test, and results with significant pleiotropy (*p* < 0.05) were excluded. Given the exploratory nature of this proteome‐wide analysis, a nominal significance threshold of *p* < 0.05 was used, consistent with previous large‐scale MR studies [11].

### 2.4. Differential Expression Analysis

Transcriptomic data for CHD were obtained from the Gene Expression Omnibus (GEO) database (https://www.ncbi.nlm.nih.gov/geo/), specifically dataset GSE179789. This study included 16 peripheral blood samples, comprising eight CHD patients and eight healthy controls [12]. The limma package was used to perform differential gene expression analysis, with *p* < 0.05 considered statistically significant. Genes identified through MR analysis that were not significantly differentially expressed or had inconsistent directions of expression were excluded from further analysis.

### 2.5. Upstream Regulation by DNA Methylation

This study employed a two‐step MR mediation analysis to investigate the regulatory effect of DNA methylation on the key gene ITGB7. Methylation quantitative trait locus (mQTL) data were obtained from the GOdMC database (http://www.godmc.org.uk/) [13]. Methylation sites related to ITGB7 were extracted, and SNPs were filtered based on the following criteria: *p* < 5e − 8, clump_kb = 10,000, and clump_*r*2 = 0.1. Ultimately, mQTL data for nine methylation sites of ITGB7 were selected as exposure variables. In the first step, the causal effect of the mQTLs on CHD was estimated (denoted as beta_*k*). In the second step, the effect of the mQTLs on ITGB7 expression (beta_*m*) and the effect of ITGB7 on CHD (beta_*n*) were calculated. The mediation effect was estimated as beta_*m* *n* = beta_*m* × beta_*n*, and the mediation proportion was calculated as beta_*p* = (beta_*m* *n*/beta_*k*) × 100*%*. A mediation proportion greater than 10% was considered indicative of a significant mediating effect [14].

### 2.6. Downstream Mechanisms of Immune Cells and Plasma Metabolites

This study used a two‐step MR mediation analysis to explore whether ITGB7 promotes CHD by regulating immune cells or plasma metabolites. Immune cell data were downloaded from the IEU Open GWAS database (https://gwas.mrcieu.ac.uk/) [15], and plasma metabolite data were obtained from the GWAS Catalog database (https://www.ebi.ac.uk/gwas/) [16]. SNPs were filtered based on the following criteria: *p* < 5e − 8, clump_kb = 10,000, and clump_*r*2 = 0.1. Ultimately, 731 immune cells and 1352 plasma metabolites were selected as exposure data. Taking immune cells as an example, in the first step, the causal effect of immune cells on CHD was estimated (denoted as beta_*n*). In the second step, the effect of ITGB7 on immune cells (beta_*m*) and the effect of ITGB7 on CHD (beta_*k*) were calculated. The mediation effect was estimated as beta_*m* *n* = beta_*m* × beta_*n*, and the mediation proportion was calculated as beta_*p* = (beta_*m* *n*/beta_*k*) × 100*%*. A mediation proportion greater than 10% was considered indicative of a significant mediating effect. The analysis of plasma metabolites followed the same procedure.

### 2.7. Statistical Methods

All statistical analyses were performed using R software (Version 4.1.6, R Foundation for Statistical Computing, Vienna, Austria), with a *p* < 0.05 considered statistically significant.

## 3. Results

### 3.1. MR Analysis Identified 17 Genes Significantly Associated With Increased CHD Risk

**Figure 2:**
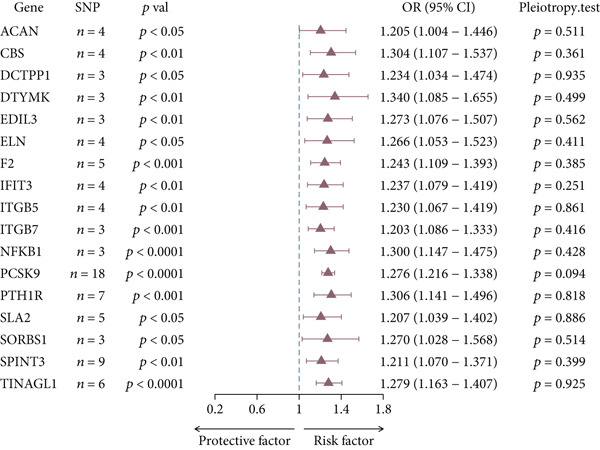
IVW model analysis of 17 genes significantly positively associated with CHD.

In this study, we used *cis*‐pQTL data for 1812 genes as exposure data and CHD as outcome data, and the results revealed that 341 genes were causally associated with CHD (S1). Horizontal pleiotropy tests showed that 23 results had significant horizontal pleiotropy (*p* < 0.05), so these were excluded. By setting OR > 1.2 to identify genes significantly positively correlated with CHD, we found that 17 genes—ACAN, CBS, DCTPP1, DTYMK, EDIL3, ELN, F2, IFIT3, ITGB5, ITGB7, NFKB1, PCSK9, PTH1R, SLA2, SORBS1, SPINT3, and TINAGL1—were significantly associated with an increased risk of CHD (Figure 2).

### 3.2. PCSK9 and ITGB7 Are Significantly Expressed in CHD Samples

We analyzed the expression of the 17 identified genes in peripheral blood samples from CHD patients and normal controls using the GSE179789 dataset. The *p* < 0.05 was set as the screening criterion. The results showed that PCSK9 and ITGB7 (Figure 3) were significantly expressed in the peripheral blood of CHD patients (*p* < 0.05).

Figure 3The differential expression of (a) ITGB7 and (b) PCSK9 in peripheral blood samples of CHD.

**Figure:**
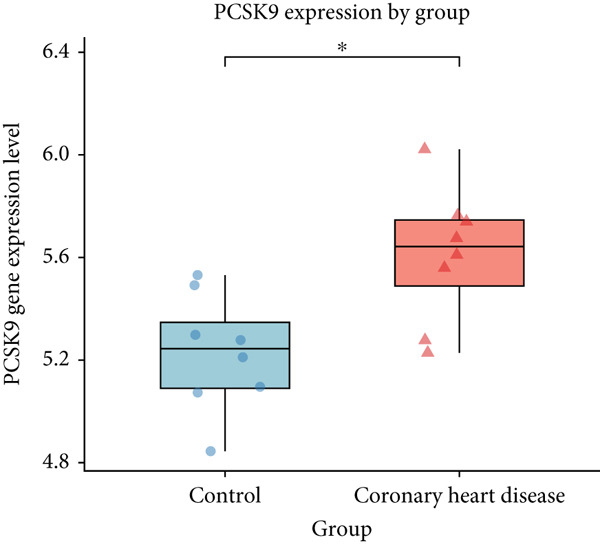
(a)

**Figure:**
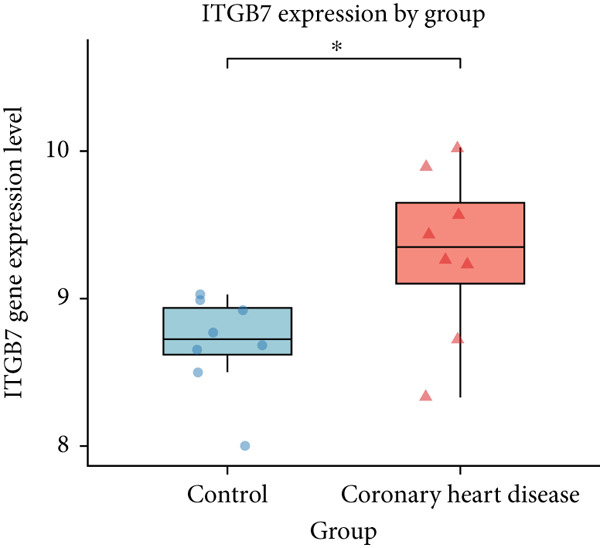
(b)

### 3.3. DNA Methylation Regulates ITGB7 to Promote CHD Progression

**Figure 4:**
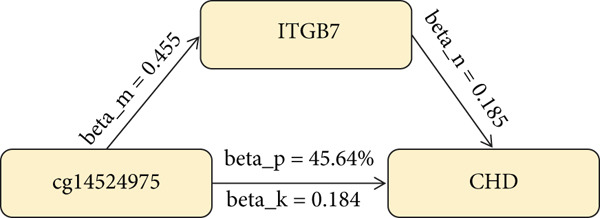
The methylation site cg14524975 regulates ITGB7 to increase the risk of CHD.

In the first step, we used nine methylation sites of ITGB7 (cg04552292, cg04972065, cg08699196, cg11510999, cg14524975, cg15889057, cg20135711, cg23029655, and cg26689077) as exposure data and CHD as the outcome data, with IVW and *p* < 0.05 as the screening criteria. The results showed that cg14524975 significantly increased the risk of CHD (OR = 1.202, 95% CI [1.103, 1.310], *p* = 2.60e − 5). In the second step, using cg14524975 as the exposure data and ITGB7 as the outcome data, the results showed that cg14524975 was significantly positively correlated with ITGB7 (OR = 1.577, 95% CI [1.070, 2.323], *p* = 0.021). Mediation analysis indicated that the DNA methylation site cg14524975 (beta_*p* = 45.64*%*) significantly regulated ITGB7 to increase the risk of CHD (Figure 4).

### 3.4. ITGB7 Regulates Immune Cells to Promote CHD Progression

**Figure 5:**
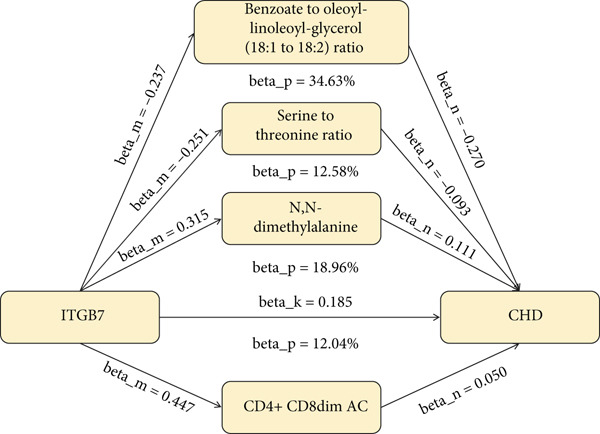
ITGB7 regulates immune cells and plasma metabolites to increase the risk of CHD.

In the first step, we used 713 immune cell types as exposure data and CHD as outcome data, with IVW and *p* < 0.05 as the screening criteria. The results revealed that 53 immune cells were causally associated with CHD (S2), with no significant horizontal pleiotropy observed. In the second step, using ITGB7 as the exposure data and the 33 immune cells identified in the first step as the outcome data, the results showed that two immune cells (CD4+ CD8dim AC and CX3CR1 on monocyte) were causally associated with CHD. Mediation analysis indicated that CD4+ CD8dim AC (beta_*p* = 12.04*%*) significantly mediated the effect of ITGB7 on CHD (Figure 5), while CX3CR1 on monocyte (beta_*p* = 5.62*%*) had a weak mediating effect.

### 3.5. ITGB7 Regulates Plasma Metabolites to Promote CHD Progression

In the first step, we used 1352 plasma metabolites as exposure data and CHD as outcome data, with IVW and *p* < 0.05 as the screening criteria. The results showed that 154 plasma metabolites were causally associated with CHD (S3), with horizontal pleiotropy tests revealing five results with significant horizontal pleiotropy (*p* < 0.05), which were excluded. In the second step, using ITGB7 as the exposure data and the 149 plasma metabolites identified in the first step as the outcome data, the results showed that eight plasma metabolites were causally associated with CHD. Mediation analysis revealed that *N*,*N*‐dimethylalanine (beta_*p* = 18.96*%*), benzoate‐to‐oleoyl–linoleoyl–glycerol (18:1 to 18:2) ratio (beta_*p* = 34.63*%*), and serine‐to‐threonine ratio (beta_*p* = 12.58*%*) significantly mediated the effect of ITGB7 on CHD (Figure 5), while *N*‐acetyl‐L‐glutamine (beta_*p* = 3.35*%*) and *N*‐delta‐acetylornithine (beta_*p* = 3.69*%*) had a weak mediating effect.

## 4. Discussion

CHD is the leading cause of cardiovascular disease–related mortality worldwide [17]. Despite significant advances in clinical interventions, its pathogenesis remains complex and not yet fully elucidated [18, 19]. In recent years, multiple genomic studies have identified susceptibility genes such as PCSK9 and LPA associated with CHD risk, providing important clues to the molecular basis of the disease [6, 7]. However, most existing studies have been limited to a single‐omics level and have lacked systematic integration of upstream epigenetic regulation and downstream immune–metabolic interactions. Consequently, the multilayer causal mechanisms underlying CHD remain insufficiently understood [8]. In this study, we established an integrated multiomics MR framework that combines DNA methylation, immune cell, and metabolite data to investigate the causal role and potential mechanisms of ITGB7 in CHD, providing new molecular evidence and potential targets for the precision prevention and treatment of cardiovascular diseases.

Through MR analysis, this study identified 17 genes significantly associated with CHD, including PCSK9 and ITGB7. PCSK9, as a well‐recognized target gene for CHD, has successfully demonstrated the potential of gene‐targeted therapy in clinical applications [20]. PCSK9 is a serine protease that degrades LDL receptors in the liver, controlling LDL levels in plasma. Inactivating PCSK9 can lower LDL levels and reduce CHD risk [21]. Two antibody‐based PCSK9 inhibitors have been successfully developed for clinical use, proving effective in lowering cholesterol levels and reducing the risk of ASCVD events, including myocardial infarction, stroke, and death, without significant adverse effects [22]. However, ITGB7, as an emerging candidate gene, has not been fully studied in CHD [23]. This study found that ITGB7 is significantly expressed in the peripheral blood of CHD patients, and its expression is closely related to CHD risk, providing strong evidence for ITGB7 as a potential therapeutic target.

ITGB7 is an integrin subunit, and previous studies have shown that elevated ITGB7 expression is closely related to the progression of multiple myeloma [24], pancreatic cancer [25], and intestinal inflammation [26]. However, the specific mechanism of ITGB7 in CHD remains unclear. DNA methylation is an important mechanism of gene expression regulation, especially in the interaction between environmental and genetic factors [27]. Changes in DNA methylation may be closely related to environmental factors, which could influence the onset of CHD in the early stages by altering the epigenetic state of genes [28]. Epigenetic editing can achieve long‐lasting therapeutic effects by silencing disease‐causing genes without altering the underlying DNA sequence [29]. In one study, an epigenetic editor was used to target the human PCSK9 gene and induce DNA methylation at the gene locus. The results showed that this approach effectively and persistently reduced circulating PCSK9 protein levels by approximately 90% while simultaneously lowering LDL‐C levels by about 70% [30]. Therefore, investigating the DNA methylation status of ITGB7 and its impact on CHD may help uncover the therapeutic potential of ITGB7.

On the other hand, immune cells [31] and plasma metabolites [32] play key roles in the development and progression of CHD, particularly during atherosclerosis formation. As a member of the integrin family, ITGB7 is involved in immune cell adhesion, migration, and inflammatory signaling. Previous studies have shown that PCSK9 can regulate T‐cell activation and expansion to mediate inflammatory responses and modulate various immune cell types [33]. In this study, ITGB7 was found to potentially regulate the activity of CD4^+^ CD8dim AC cells in CHD. Aberrant expression of ITGB7 may enhance immune cell infiltration and vascular inflammation, thereby promoting atherosclerotic progression and increasing CHD risk [34]. Thus, investigating the immune regulatory mechanisms mediated by ITGB7 may provide new insights for immunotherapy in CHD. In addition, this study identified significant causal associations between ITGB7 and several plasma metabolites, including *N*,*N*‐dimethylalanine, the benzoate‐to‐oleoyl–linoleoyl–glycerol ratio, and the serine‐to‐threonine ratio, which showed strong mediation effects. These metabolites are critically involved in lipid metabolism, amino acid metabolism, and oxidative stress, processes known to contribute to atherosclerosis and endothelial dysfunction [35]. The interactions between genes and metabolites form a complex metabolic network [36], suggesting that ITGB7 may play a pivotal role in CHD through metabolic regulation. Further studies exploring the mechanistic links between ITGB7 and these metabolites may reveal novel diagnostic and therapeutic targets for CHD.

Although this study revealed the potential role of ITGB7 in CHD, several limitations should be noted. First, the main datasets used in this study were obtained from publicly available databases. Although the sample sizes were relatively large, further experimental validation is still required. Second, the association between ITGB7 and CHD needs to be confirmed through larger clinical cohorts and functional experiments. Third, although MR analysis can effectively reduce confounding bias, it may still be influenced by residual horizontal pleiotropy, linkage disequilibrium (LD), and population stratification. In this study, we assessed pleiotropy using the MR–Egger intercept test and strictly controlled LD during the selection of instrumental variables to minimize potential bias. However, since the UKB‐PPP and FinnGen datasets used in this study are primarily based on European populations, the generalizability of these findings to other ethnic groups (e.g., East Asian populations) remains uncertain. Future studies should replicate the analysis in multiethnic GWAS datasets and combine experimental and clinical validation to enhance the robustness and external validity of the results.

This study suggests that ITGB7 may serve as a potential therapeutic target for CHD, although its clinical translation requires further investigation. As an important member of the integrin family, ITGB7 plays a key role in immune cell adhesion, migration, and inflammatory signaling, indicating its potential as a novel target for anti‐inflammatory or immunomodulatory therapy. Meanwhile, its stable expression pattern in peripheral blood suggests potential diagnostic and prognostic biomarker value, which could be utilized for early CHD risk assessment and monitoring of therapeutic responses. Future studies should further validate the functional role of ITGB7 through animal experiments and clinical samples, evaluate the feasibility of interventions targeting this pathway, and explore its potential roles in other cardiovascular diseases. In addition, integrating multiomics data with clinical information to comprehensively elucidate the regulatory network of ITGB7 may provide new molecular insights and targeted strategies for the precision diagnosis and treatment of cardiovascular diseases.

## 5. Conclusion

This study, through a multiomics research strategy, reveals the potential role of ITGB7 in the development of CHD, particularly its promotive effects on CHD through the regulation of DNA methylation, immune cells, and plasma metabolites. As a novel molecular target, ITGB7 provides new ideas and directions for the treatment of CHD. Further research is expected to advance the clinical application of ITGB7 in cardiovascular diseases, thereby providing a new theoretical basis and practical guidelines for the precision treatment of CHD.

## Ethics Statement

The authors have nothing to report.

## Consent

The authors have nothing to report.

## Conflicts of Interest

The authors declare no conflicts of interest.

## Author Contributions

Junchi Guo: writing (original draft), visualization, data curation, and software. Fang Huang: writing (original draft) and visualization. Peihan Lu: writing (original draft) and software. Meijuan Lu: writing (review and editing), methodology, funding acquisition, and conceptualization.

## Funding

This study was funded by the Tianjin Traditional Chinese Medicine and Integrated Traditional Chinese and Western Medicine Research Project, 2023027.

## Supporting information


**Supporting Information** Additional supporting information can be found online in the Supporting Information section. S1: Genes significantly associated with CHD. S2: Immune cells significantly associated with CHD. S3: Plasma metabolites significantly associated with CHD.

## Data Availability

The data that support the findings of this study are available from the corresponding author upon reasonable request.
